# ﻿New relictual genera in Cyrtoquediini and Indoquediini (Coleoptera: Staphylinidae: Staphylininae)

**DOI:** 10.3897/zookeys.1076.73103

**Published:** 2021-12-09

**Authors:** Adam J. Brunke

**Affiliations:** 1 Agriculture and Agri-Food Canada, Canadian National Collection of Insects, Arachnids and Nematodes, 960 Carling Avenue, Ottawa, Ontario, Canada Canadian National Collection of Insects Ottawa Canada

**Keywords:** Rove beetles, Nearctic, Oriental, taxonomy, systematics, identification keys

## Abstract

*Sundaquedius***gen. nov.** (Cyrtoquediini) and *Fluviphirus***gen. nov.** (Indoquediini) are described from southeast Asia and western North America, respectively, resulting in the new combinations *Sundaquediusnigropolitus* (Cameron) and *Fluviphiruselevatus* (Hatch). *Sundaquediusabbreviatus***sp. nov.** is described from Vietnam. The phylogenetic positions of these genera within Staphylininae are supported by morphology and recently published phylogenomic evidence. New keys to the world genera of Cyrtoquediini and Indoquediini are provided. A new country record for *Alesiellalineipennis* (Cameron) is provided for Thailand, based on the first available specimen in more than 100 years.

## ﻿Introduction

The tribe Quediini (formerly Quediina, see Materials and methods) was previously a convenient dumping ground for plesiomorphy-rich taxa in Staphylininae, and its largest genus, *Quedius*, was the destination for most of these species (summarized by Solodovnikov 2006a). Numerous phylogenetic analyses using both morphological and molecular evidence have sought to identify monophyletic lineages within this heterogeneous assemblage and a number of higher taxa have been created to represent these separately from Quediini and *Quedius* (e.g., Chatzimanolis et al. 2010; Brunke et al. 2016, 2019, 2021). With a diverse monophyletic core of Quediini largely now delimited, Brunke et al. (2021) assembled a phylogenomic dataset to resolve the position of remaining Staphylininae*incertae sedis* taxa and identify any remaining *Quedius* species that may belong to other tribes. These analyses revealed that two species of *Quedius*, *Q.elevatus* (Hatch) from western North America and an undescribed species from Vietnam, very closely related to *Q.nigropolitus* (Cameron) from East Java, clearly belonged to Indoquediini and Cyrtoquediini, respectively and represented undescribed genera. These species were also found to share morphological synapomorphies with other members of their tribe, though they were geographically quite distant from their closest relatives. Several Staphylininae*incertae sedis* genera were also recovered by Brunke et al. (2021) as Cyrtoquediini and Indoquediini, resulting in significant changes to the composition and morphological diagnoses of these tribes. This paper aims to formally describe and illustrate these two genera, and provide the first keys to the world genera of Indoquediini and Cyrtoquediini as recently revised by Brunke et al. (2021).

## ﻿Materials and methods

### ﻿Depositories:

**cHay** Personal collection of Y. Hayashi, Kawanishi City, Japan

**NMUK**Natural History Museum, London, U.K. (M. Geiser, M. Barclay)

**CNC** Canadian National Collection of Insects, Arachnids and Nematodes, Ottawa, Ontario, Canada

**ZIN**Zoological Institute, Russian Academy of Sciences, St. Petersburg, Russia (via A. Solodovnikov)

### ﻿Specimen data

Type label data are given verbatim, with labels separated by “/” and comments indicated in square brackets. Non-type label data were standardized to improve clarity. Specimens were georeferenced using Google Earth or Google Maps.

### ﻿Microscopy, illustration, and photography

All specimens were examined dry using a Nikon SMZ25 stereomicroscope. Genitalia and terminal segments of the abdomen were dissected and placed in glycerin filled vials, pinned with their respective specimens. Line illustrations were made from standard images and then digitally inked in Adobe Illustrator CC-2020. All imaging, including photomontage was accomplished using a motorized Nikon SMZ25 microscope and NIS Elements BR v4.5. Photos were post-processed in Adobe Photoshop CC-2020.

### ﻿Measurements and character variability

All measurements were made using a live measurement module within NIS Elements BR v4.5. Measurements were taken as listed below, but only proportional (HW/HL, PW/PL, EW/EL, PW/HW) and forebody measurements are stated directly in descriptions. Total body length is generally difficult to standardize for Staphylinidae and was not measured due to the contractile nature of the abdomen.

**HL** Head Length, at middle, from the anterior margin of frons to the nuchal ridge.

**HW** Head Width, the greatest width, including the eyes.

**PL** Pronotum Length, at middle.

**PW** Pronotum Width, greatest width.

**EL** Elytral Length, greatest length taken from level of the anterior most large, lateral macroseta to apex of elytra. EL approximates the length of the elytra not covered by the pronotum and therefore contributing to the forebody length.

**EW** Elytral Width, greatest width.

**Forebody**HL + PL + EL.

### ﻿Terminology and higher classification

Morphological terminology follows that of Brunke et al. (2019, 2021). Recent phylogenetic studies (Tihelka et al. 2020; Żyła and Solodovnikov 2020) have proposed alternate solutions for the limits of subfamily Staphylininae (i.e., including or excluding the xantholinine group, Arrowinini and genus *Coomania* Cameron). In the stricter sense (sensu Żyła & Solodovnikov 2020), the xantholinine group, *Arrowinus*+*Platyprosopus*, *Coomania* and the remaining Staphylininae are each treated at the subfamily level with diagnostic character states. The system proposed by Żyła & Solodovnikov (2020) is preferred here for its greater diagnostic value and therefore Cyrtoquediini, Quediini, etc. are treated as tribes of Staphylininae herein.

## ﻿Taxonomy

### Staphylininae Latreille, 1802

#### ﻿ Cyrtoquediini

Taxon classificationAnimaliaColeopteraStaphylinidae

Brunke & Solodovnikov, 2016

2EA08D86-F908-5E09-BFA3-18FAF72F74D5

##### Diagnosis.

Cyrtoquediini (as recently redefined by Brunke et al. (2021)) can be recognized among other Staphylininae based on the following combination of characters: microsculpture on disc of head and pronotum absent; obvious presence of both posterior frontal and basal punctures (Brunke et al., 2019: fig. 1); profemora with apical row of lateroventral spines (near joint with protibia) (Brunke et al. 2021: fig. 8B); protibia without subapical notch (Brunke et al. 2021: fig. 8C); metatarsomeres 1–4 flattened and trapezoidal, not elongate and cylindrical. Most genera in Cyrtoquediini can also be recognized by the unique row of coarse, impressed setose punctures on the elytral epipleuron (Brunke et al. 2016: fig. 4).

#### ﻿Key to world genera of Cyrtoquediini

**Table:** 

1	Last segment of maxillary and labial palpi strongly dilated to a truncate apex; mandibles without teeth; West Palaearctic	***Astrapaeus* Gravenhorst (*A.ulmi* (Rossi))**
–	Last segment of maxillary and labial palpi not strongly dilated to truncate apex; mandibles with at least one tooth each; Nearctic, Neotropical, East Palaearctic and Oriental Regions	**2**
2	Eyes relatively small, eye no more than 1.5 × longer than temple	**3**
–	Eyes relatively large, eye nearly 3 × as long as temple or larger, temple usually very small	**6**
3	Dorsal head and pronotum entirely covered in fine setae; inside termite nests (*Nasutitermes* Dudley); known only from South America; 2 spp., key in Solodovnikov (2006b)	***Sedolinus* Solodovnikov**
–	Dorsal head and pronotum glabrous except for macrosetae	**4**
4	Antennomeres 1–3 without tomentose pubescence; body small (< 6.0 mm); ventral head with infraorbital ridge extending to base of mandibles; mesoscutellum without micropunctures; associated with fungusy rotting wood in older stages of decomposition (Hu and Bogri 2020); 5 spp., key in Hu et al. (2013)	***Quwatanabius* Smetana**
–	Antennomeres 1–4 without tomentose pubescence; body large (approx. 1 cm or more); ventral head with infraorbital ridge restricted to basal third of head length or less; mesoscutellum with micropunctures; associated with early, fermenting stages of decay such as rotting *Agave* or *Myrtillocactus* (Navarrete et al. 2002), or under the bark of sappy logs	**5**
5	Abdomen bicolored red (basal three segments) and black (apical two segments); mesoventrite with horn-like projection; Oriental Region, known from Myanmar and northern Thailand	***Alesiella* Brunke & Solodovnikov (*A.lineipennis* (Cameron))**
–	Abdomen uniformly dark; mesoventrite without horn-like projection; Neotropical Region, known from Mexico to Costa Rica; 2 spp., notes in Brunke and Solodovnikov (2013)	***Quediomacrus* Sharp**
6	Elytra with irregular, coarse and asetose macropunctures; antennomeres 1–5 without tomentose pubescence; 78 spp., keys in Brunke and Solodovnikov (2014), Brunke (2017)	***Bolitogyrus* Chevrolat**
–	Elytral with macropunctures setose, organized in rows, surface sometimes with scattered micropunctures; antennomeres 1–3 without tomentose pubescence (tomentose pubescence sometimes partly missing on antennae 4)	**7**
7	Head with two or more parocular punctures (Fig. 1B, E); infraorbital ridge incomplete, not reaching base of mandible; pronotum with at least two punctures in dorsal row (Fig. 1C, F); Oriental Region	***Sundaquedius* Brunke, gen. nov.**
–	Head with only one parocular puncture; infraorbital ridge complete, reaching base of mandible; pronotum with only one puncture in dorsal row (marginal puncture); Nearctic and Neotropical Regions	**8**
8	Head, pronotum and elytra distinctly flattened; meso- and metatarsomeres markedly bilobed, transverse; tarsomere 4 reaching half the length of tarsomere 5; occurs under the bark of decaying trees (Brèthes 1900); known only from the Buenos Aires area, Argentina	***Parisanopus* Brèthes (*P.castaneipennis* Brèthes)**
–	Forebody distinctly convex; meso- and meta tarsomeres less strongly bilobed, not transverse; tarsomere 4 not reaching half the length of tarsomere 5; southern half of Nearctic Region and broadly distributed within the Neotropical Region; 23 extant spp., listed in Brunke et al. (2016)	***Cyrtoquedius* Bernhauer**

#### ﻿ Alesiella lineipennis

Taxon classificationAnimaliaColeopteraStaphylinidae

(Cameron, 1932)

20E1B7B6-3D6E-5803-A70E-990EA4551A91

Quedius (Quedionuchus) lineipennis Cameron, 1932
Quedius lineipennis Cameron: Smetana 1988
Alesiella lineipennis (Cameron): Brunke and Solodovnikov 2013

##### Type locality.

Mogok [= Ruby Mines], Mandalay, Myanmar

##### Non-type material.

**Thailand: Chiang Rai**: Wiang Pa Pao District [no specific locality], 17–21.V.2015, K. Takahashi (1 male, aedeagus missing, cHay).

##### Diagnosis.

Only one species of *Alesiella* is known and can be recognized by characters in the above key to genera. The specimen from Thailand does not differ from the type material (previously studied by the author), though the aedeagus was lost during mounting (Y. Hayashi, pers. comm.).

##### Distribution.

Myanmar and Thailand (new country record).

##### Bionomics.

Nothing is known about this species’ microhabitat preferences but it probably occurs under the bark of dead trees in the earlier fermentation states of decay, as does its sister group *Quediomacrus*.

##### Comments.

The above specimen is a new record of the genus and species from Thailand, and represents the first available material in more than 130 years (since 1890). The above record also indicates that the species is certainly still extant and rather widespread, though its elevational range remains unknown. Although the type series only bears the information ‘Ruby Mines, Doherty’, the diaries of William Doherty (reproduced in Hartert 1901) indicate that they were collected somewhere along the route (1150–1800 m) between the towns of Mogok (= ‘Ruby Mines’) and Bernardmyo, during 1890. This montane rainforest elevational range is compatible with the general locality of the specimen from Thailand.

#### ﻿ Sundaquedius

Taxon classificationAnimaliaColeopteraStaphylinidae

Brunke gen. nov.

496DB274-96DD-55CD-9899-7BC808D87444

http://zoobank.org/B1952B22-6F67-4717-9C69-74F8C5E5498D

Figs 1A–F , 2A–G

##### Type species.

*Sundaquediusabbreviatus* Brunke, sp. nov.

##### Etymology.

The generic name refers to the Sunda Plate and *Quedius*, with which members of this genus and closely related genus *Cyrtoquedius* were associated with for a long time. Much of the Sunda Plate is currently below sea level but had connected terrestrial species on Borneo, Sumatra, Java and the present southeast Asian mainland in multiple episodes, from about the Eocene to as recently as the Pleistocene (e.g., Inger and Voris 2001). Noun in apposition.

##### Diagnosis.

**Figure 1.**
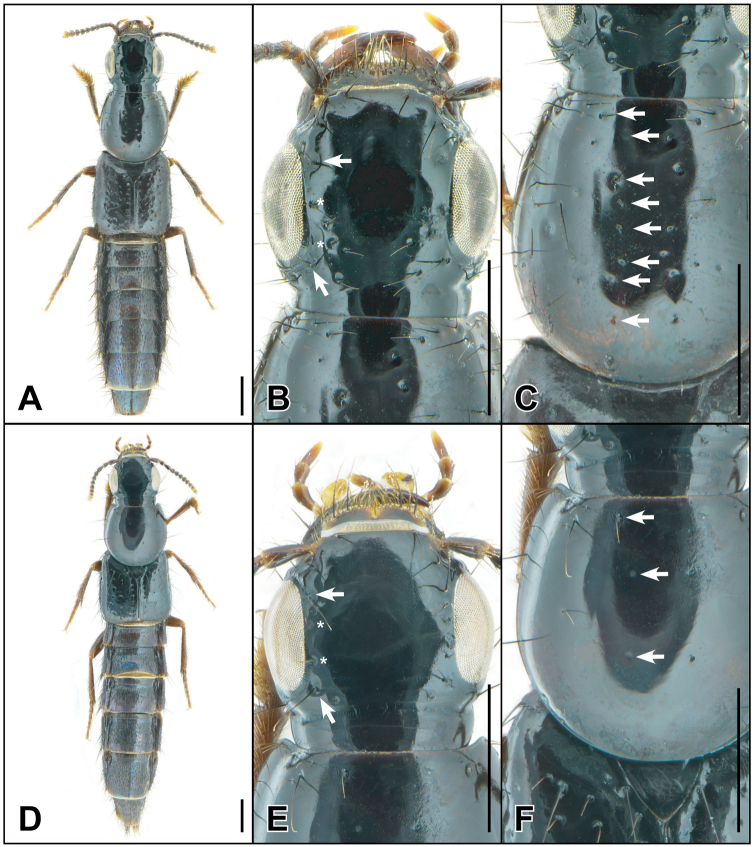
*Sundaquedius* Brunke **A–C***S.nigropolitus* (Cameron) **D–F***S.abbreviatus* Brunke **A, D** habitus **B, E** dorsal head, arrows indicating anterior and posterior frontal punctures, asterisks indicating parocular punctures **C, F** dorsal pronotum, arrows indicating punctures of the dorsal row. Scale bars: 1 mm.

**Figure 2.**
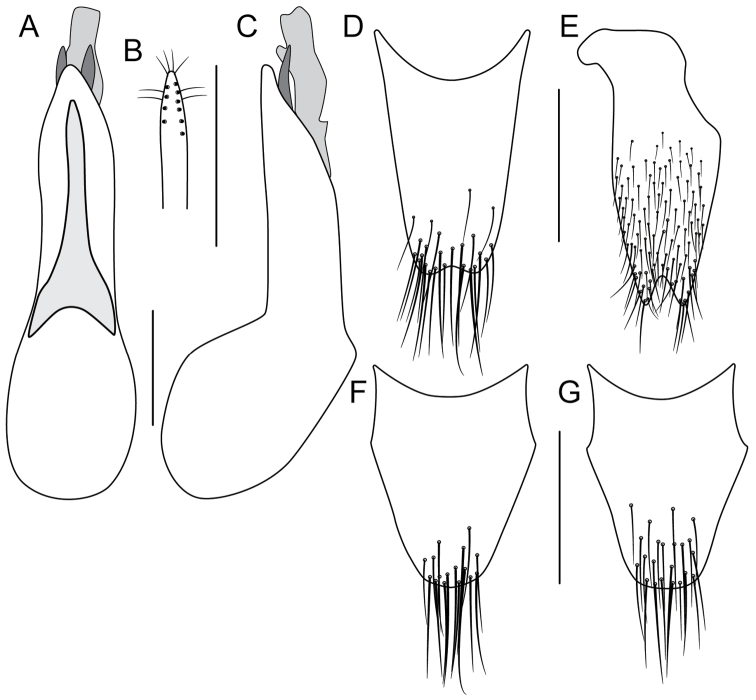
*Sundaquedius* Brunke **A–F***S.abbreviatus* Brunke **G***S.nigropolitus* (Cameron) **A** aedeagus, ventral view **B** apex of paramere, underside **C** aedeagus, lateral view (paramere removed) **D** male tergite X **E** male sternite IX **F, G** female tergite X. Scale bars: 0.25 mm (**A–C**); 0.5 mm (**D–G**).

Among other Oriental Cyrtoquediini, *Sundaquedius* is easily recognized by a combination of the large eyes (more than 3 × as long as temples) (Fig. 1B, E), incomplete infraorbital ridge and elytra with rows of setose punctures. It can be distinguished from its putative close relatives *Cyrtoquedius* and *Parisanopus* by any one of: more than one puncture in the dorsal row of the pronotum (Fig. 1C, F), two or more parocular punctures on the head (Fig. 1B, E), the incomplete infraorbital ridge and presence of peg setae on the paramere (Fig. 2B).

##### Description.

With the character states of Cyrtoquediini (see Brunke et al. 2021) and the following: head with basal puncture present but not doubled; two or three parocular punctures present; antennae non-geniculate, antennomeres 1–3 sparsely pubescent and without tomentose pubescence, antennomere 4 with some tomentose pubescence but much sparser than 5; labrum with two usual lobes and moderately incised median emargination; apical maxillary and labial palpi fusiform, apical labial palpomere with sparse, short setae; mandibles slender in apical half and markedly broad in basal half, bearing a single proximal tooth; gular sutures convergent, separate but running extremely close in basal half; infraorbital ridge/nuchal ridge incomplete, reaching ~ 1/3 to 1/2 the distance to mandible base; pronotum strongly convex, non-explanate and slightly elongate, with 2–8 punctures in the dorsal row, ‘second’ puncture present; basisternum with pair of macrosetae at middle; mesoscutellum glabrous and without micropunctures; disc of elytra without microsculpture and glabrous, except for three rows of coarse setose macropunctures (one sutural, two discal), rows slightly disorganized due to extra punctures in *S.nigropolitus*; elytra with epipleuron bearing row of coarse, setose macropunctures, epipleuron with additional rows and clusters of coarse setae; epipleural margin not thickened; mesocoxae contiguous; metatibia spinose, with three spines on outer face, inner face without spines; pro- and metatarsomeres with setae on disc, setae not restricted to margins; metatarsomere 4 with ventral setae distinctly interrupted medially and removed from apical margin; abdominal tergite IV with impression but punctures only slightly more impressed, not markedly coarser in impression (as in *Bolitogyrus*); abdominal sternite III with basal transverse line sharply produced posteriad forming an acute angle at middle; abdominal sternite IV with basal transverse line not produced; aedeagus with single fused paramere bearing well-developed peg setae, internal sac with ventral, paired copulatory sclerites, with an additional sclerotized structure similar to dorsal copulatory piece, but singular, and more weakly sclerotized compared to *Cyrtoquedius* or *Parisanopus*, and held within spinose internal sac.

##### Distribution.

*Sundaquedius* is presently known only from central Vietnam and East Java but likely occurs at medium elevations across southeast Asia, west of Wallace’s line.

##### Bionomics.

Nothing is known about the bionomics of this genus, except that both species were collected in lower montane forests (700–1500 m). *Sundaquedius* might be collected by sifting moist litter, like many species of the related genus *Cyrtoquedius*.

##### Comments.

In recent phylogenomic analyses, *Sundaquedius* was recovered as the sister group of Nearctic/Neotropical genus *Cyrtoquedius* with high support, though few genera of Cyrtoquediini were included in the taxon sample (Brunke et al. 2021). *Sundaquedius* is probably most closely related to *Cyrtoquedius*, or perhaps *Cyrtoquedius*+*Parisanopus*, based on morphological similarity (see above key).

#### ﻿Key to species of *Sundaquedius* Brunke, gen. nov.

**Table:** 

1	Pronotum with 2 or 3 punctures in the dorsal row (Fig. 1F); head without additional macropunctures (Fig. 1E); elytra with discal rows organized and without scattered punctures between (Fig. 1D); Vietnam	***S.abbreviatus* Brunke sp. nov.**
–	Pronotum with 7 or 8 punctures in the dorsal row (1C); head with additional macropunctures (Fig. 1B); elytra with discal rows slightly disorganized, with additional scattered punctures between (Fig. 1A); East Java	***S.nigropolitus* (Cameron)**

#### ﻿ Sundaquedius nigropolitus

Taxon classificationAnimaliaColeopteraStaphylinidae

(Cameron) comb. nov.

35316AE5-F203-5925-8E78-B9EF35BD318C

Figs 1A–C , 2G

Quedius (Sauridus) nigropolitus Cameron, 1937
‘Quedius’ nigropolitus Cameron: Brunke et al. (2021) (in undescribed genus of Cyrtoquediini)

##### Type locality.

Blawan [sometimes ‘Belawan’], Ijen Plateau [no specific locality, ca. -7.98, 114.17], Bondowoso Regency, East Java, Indonesia.

##### Type material.

***Holotype*** (female, NMUK): Type [circular label with red border] / leg H. Lucht, K.O. Blawan, Idjen-Plateau [Ijen Plateau] Java, 900-1500 mr., 12.I.1934 [printed label] / Q.nigropolitus Type Cameron [handwritten] / M. Cameron, Bequest, B.M. 1955-147. [printed label] / AJB0001486 [printed label] / HOLOTYPE *Quediusnigropolitus* Cameron, det. A. Brunke 2021 [red label] / *Sudaquediusnigropolitus* (Cameron) [white label], det. A. Brunke 2021

##### Diagnosis.

*Sundaquediusnigropolitus* can be easily recognized by the dorsal rows of the pronotum, which have seven or eight punctures in each row. The only other known species is allopatric.

##### Redescription.

Measurements ♀ (n = 1): HW/HL 1.23; PW/PL 0.94; EW/EL 1.06; PW/HW 1.13; forebody length 4.8 mm.

Body highly glossy, entirely black, except for yellowish brown apical antennomere, tarsi and apical maxillary and labial palpomeres, abdomen with iridescent sheen ranging from violet to blue.

Head distinctly transverse, with two or three additional punctures mediad of posterior frontal puncture (Fig. 1B). Antennomeres 1–3 elongate, 4 subquadrate, 5 and 6 weakly transverse, 7–10 strongly transverse, 10 ~ 2 × as wide as long. Pronotum slightly elongate with seven or eight punctures in the dorsal row, with additional groups of scattered punctures between dorsal and sublateral rows, and between sublateral row and lateral margin (Fig. 1C). Elytra transverse, with additional scattered punctures between the two discal rows (Fig. 1A). Abdominal tergites III–IV with distinct basal impression, tergites III–V with median or medioapical glabrous or sparsely punctate areas, these areas successively becoming smaller toward the apex; abdominal punctures generally coarse, nearly all separated by at least their diameters; tergites with exceedingly fine and dense microsculpture of transverse waves.

Male unknown. Female with tergite X triangular, with slightly narrowed but broadly rounded apex, apical half with many long setae (Fig. 2G).

##### Distribution.

Known only from the type locality in East Java, which is at the northern edge of the plateau.

##### Bionomics.

Nothing is known about this species’ microhabitat preferences.

##### Comments.

The holotype of this species was one of the few specimens included from East Java in Cameron’s (1937) ‘Fauna Javanica’. This region is still extremely poorly collected for Staphylinidae, even more so than West or Central Java.

#### ﻿ Sundaquedius abbreviatus

Taxon classificationAnimaliaColeopteraStaphylinidae

Brunke sp. nov.

B03D9F4F-A991-5D55-B14A-A316150212E5

http://zoobank.org/CBC36A0B-D8DC-46C0-BB10-84D68131930E

Figs 1D–F , 2A–F

##### Type locality.

35 km north of An Khê, near Buôn Lưới village, Gia Lai, Vietnam [ca. 14.32, 108.58].

##### Type material.

***Holotype*** (male, CNC): Vietnam, 35 km N An Khe, Buon Luoi, 2.VII.1984 / AJB0001487 [white label] / HOLOTYPE *Sundaquediusabbreviatus* Brunke, des. A. Brunke 2021 [red label] ***Paratypes*** (5, ZIN): same data as the holotype but with labels: PARATYPE *Sundaquediusabbreviatus* Brunke, des. A. Brunke 2021 [yellow label]. Identifiers: AJB0001334, AJB0001488–1491

##### Etymology.

The species epithet means ‘shortened’ or ‘reduced’, and refers to the shorter dorsal rows of punctures on the pronotum compared to *S.nigropolitus*.

##### Diagnosis.

*Sundaquediusabbreviatus* can be distinguished by the presence of only two or three punctures in the dorsal row of the pronotum.

##### Description.

Measurements. Male (n = 2): HW/HL 1.30–1.35; PW/PL 1.06–1.08; EW/EL 1.22–1.24; PW/HW 1.14–1.19; forebody length 4.9–5.4 mm. Female (n = 4): HW/HL 1.25–1.29; PW/PL 1.03–1.10; EW/EL 1.13–1.15; PW/HW 1.21–1.23; forebody length 4.7–5.0 mm.

Similar to *S.nigropolitus* and differing only in the following: antennomeres dark except apical three segments paler, becoming successively paler to antennal apex; maxillary and labial palpi paler, entirely medium reddish brown; head, without additional punctures between named punctures, distinctly transverse, more so in males, head also broader relative to pronotum in males; antennae overall more robust, with apical segments less strongly transverse; pronotum slightly to distinctly transverse, with two or three punctures in the dorsal row, third puncture, when present, smaller, sometimes rudimentary and without seta; elytra more transverse than in *S.nigropolitus*, and even more so in males, with two discal rows and without scattered additional punctures; abdominal tergites III and IV with distinct impressions, V with only vague impression; abdominal punctation slightly denser but punctures generally still well separated.

Male with sternite VII broadly but shallowly emarginate; sternite VIII with slightly deeper emargination and distinct, triangular impressed and glabrous area; tergite X elongate, with distinct shallow emargination, with many long setae at apex (Fig. 2D); sternite IX with bulky, asymmetrical base, apex deeply emarginate (Fig. 2E); median lobe of aedeagus in ventral view subparallel sided, narrowing to rounded, acute apex, paramere with broad base, becoming slender to strongly acute apex (Fig. 2A); median lobe in lateral view with nearly straight ventral face, with short, rounded apical part (Fig. 2C); apex of paramere with short, sparse paired row of marginal peg setae (Fig. 2B); aedeagus with ventral paired copulatory sclerites broadest at base and strongly narrowed to sharp apex. Female tergite X similar to that of *S.nigropolitus* but with slightly narrower apex (Fig. 2F).

##### Distribution.

Known only from the type locality in the central highlands of Vietnam.

##### Bionomics.

Nothing specific is known about this species but the type locality is at approximately 700–800 m, so this species likely occurs elsewhere in lower montane forests of central Vietnam and possibly adjacent Cambodia.

#### ﻿ Indoquediini

Taxon classificationAnimaliaColeopteraStaphylinidae

Brunke & Solodovnikov, 2016

B96953A8-61FA-5081-ADC1-0AABFFC4BB27

##### Diagnosis.

Indoquediini (as recently redefined by Brunke et al. (2021)) can be recognized among other Staphylininae by the combination of: head with obvious presence of both posterior frontal and basal punctures (Brunke et al. 2019: fig. 1); protibiae subapically with distinct and unique notch (Brunke et al. 2021: fig. 8C); all antennomeres longer than wide.

#### ﻿Key to world genera of Indoquediini

**Table:** 

1	Head with interocular punctures present on frons (Brunke et al. 2019: fig. 1); eyes small, less than half the length of temples; pronotum with four punctures in dorsal row; empodial setae absent; Nepal, Pakistan and China	***Strouhalium* Scheerpeltz (*S.gracilicorne* Scheerpeltz)**
–	Head without interocular punctures on frons; eyes large, clearly longer than temples; pronotum with two punctures in dorsal row; empodial setae present; western Nearctic, East Palaearctic and Oriental Regions	**2**
2	Head and pronotum without microsculpture, highly glossy; head with bulging eyes occupying nearly all of lateral head; East Palaearctic and Oriental Regions; 39 spp., listed by Brunke et al. (2016)	***Indoquedius* Blackwelder**
–	Head and pronotum with meshed microsculpture creating dull (especially head) appearance; head with eyes smaller and less convex, occupying ~ 2/3 of lateral head (Fig. 3A); western Nearctic	***Fluviphirus* Brunke, gen. nov. (*F.elevatus* (Hatch))**

#### ﻿ Fluviphirus

Taxon classificationAnimaliaColeopteraStaphylinidae

Brunke gen. nov.

E50B322C-5347-585B-901B-F70A83A4E7CE

http://zoobank.org/9E8CF744-6B68-484A-A4D1-4003CF0D291F

Fig. 3A–G

##### Type species.

*Fluviphiruselevatus* (Hatch), comb. nov.

##### Etymology.

The generic name is a combination of the Latin word *fluvium* (river, stream) and *Raphirus* (a subgenus of *Quedius*), where the only species of *Fluviphirus* was previously classified and to which it bears a superficial resemblance. Noun in apposition.

##### Diagnosis.

Among other Indoquediini, *Fluviphirus* is easily recognized by the combination of meshed microsculpture on the forebody and the absence of interocular punctures on the head. It is also the only genus of Nearctic Indoquediini.

##### Description.

**Figure 3.**
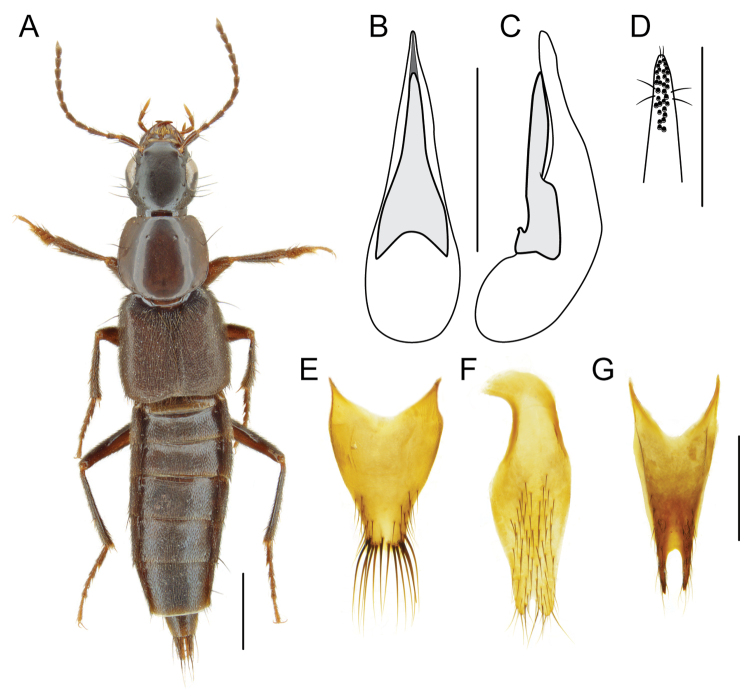
*Fluviphiruselevatus* (Hatch) **A** habitus **B** aedeagus, ventral view **C** aedeagus, lateral view **D** apex of paramere, underside **E** male tergite X **F** male sternite IX **G** female tergite X. Scale bars: 1 mm (**A**); 0.5 mm (**B–C, E–G**); 0.25 mm (**D**).

With the character states of Indoquediini (see Brunke et al. 2021) and the following: disc of head and pronotum with meshed microsculpture; eyes moderately convex, not strongly bulging, large, distinctly larger than temples (Fig. 3A); head with single basal puncture, interocular punctures absent, temples with numerous smaller punctures, with single parocular puncture; antennomere 3 with dense but not tomentose pubescence; apical maxillary palpomere glabrous; penultimate labial palpomere with brush of dense setae (but sparser than that of *Indoquedius*); pronotum with two punctures in dorsal row, ‘second’ puncture present (Fig. 3A); postcoxal process fused across inferior marginal line; elytra with sub-basal ridge reduced to horizontal fragment, with evidence of mesoscutellar collar; humeral spines absent; protibia without lateral spines (Fig. 3A); metatibia with only two thin spines on outer face (Fig. 3A); pretarsi of all legs with one pair of empodial setae; abdominal sternite III with basal transverse carina produced posteriad at a sharp angle.

##### Distribution.

Western North America, broadly distributed along the western cordilleras at a variety of elevations.

##### Bionomics.

The single species of *Fluviphirus* is strongly associated with debris along the margins of rivers and larger creeks.

##### Comments.

Smetana (1971a) placed *Q.elevatus* in its own species group as it was “quite isolated within the subgenus [Quedius (Raphirus)]”. The subgenus Raphirus remains a convenient dumping ground for many unrelated taxa (Brunke et al. 2021) because of its broad definition, with many plesiomorphic character states, including the absence of certain features typical of other clades within Quediini. Recent phylogenomic analyses recovered *Q.elevatus* as a member of Indoquediini, as the sister group of either *Strouhalium* (coalescent analyses) or *Indoquedius* (concatenated analyses). *Fluviphirus* shares a number of character states with both genera but more densely sampled, total evidence analyses are needed to determine its sister group.

#### ﻿ Fluviphirus elevatus

Taxon classificationAnimaliaColeopteraStaphylinidae

(Hatch) comb. nov.

EB0F25A8-0694-5B9B-8CD3-0A0AB2C937C6

Fig. 3A–G

Quedius (Sauridus) elevatus Hatch, 1957
Quedius (Raphirus) elevatus Hatch: Smetana 1971a, b (subgenus Raphirus, elevatus species group)
‘Quedius’ elevatus Hatch: Brunke et al. 2021 (in undescribed genus of Indoquediini)

##### Type locality.

Snoqualmie, Washington, United States.

##### Type material.

The type material of this distinctive species was not examined.

##### Non-type material.

**Canada: British Columbia**: 8 mi W Creston, ex. river debris, 10.VI.1968, J.M. Campbell & A. Smetana (8, CNC); 20 mi E Hope, ex. river debris, 3.VI.1968, J.M. Campbell & A. Smetana (1, CNC); 4 mi W Midway, ex. river debris, 6.VI.1968, J.M. Campbell & A. Smetana (6, CNC); 16 mi W Osoyoos, ex. river debris, 5.VI.1968, J.M. Campbell & A. Smetana (1, CNC); Paulson, beaver house, 7.VI.1968, J.M. Campbell & A. Smetana (1, CNC); 4 mi W Rossland, 9.VI.1968, J.M. Campbell & A. Smetana (2, CNC); 2 mi S Salmo, ex. river debris, 9.VI.1968, J.M. Campbell & A. Smetana (3, CNC); 2 mi E Burnt Flats [Burnt Flat Junction], 9.VI.1968 (2, CNC). **United States: California**: *Marin Co.*, Lagunitas Creek at Tocaloma, 18.III.1983, A. Smetana (17, CNC); same except 19.III.1983 (6, CNC). **Oregon**: Union Co., Blue Mts., Phillips Creek Road, 9 km NW Elgin (2, CNC).

##### Diagnosis.

As given above for the genus.

##### Redescription.

The species was redescribed by Smetana (1971a) but this is here supplemented with additional characters specific to the male and female: male with sternite VII unmodified; sternite VIII with broad shallow emargination; tergite X constricted in apical half, with weakly emarginate apex, apical half with short fine setae on lateral parts of disc and strong, coarse setae along apical margin (Fig. 3E); sternite IX with moderately slender, asymmetrical base, elongate with deep and narrow emargination (Fig. 3F); median lobe of aedeagus in ventral view narrowed to sharp apex, apical portion with longitudinal median excavation (Fig. 3B), paramere with broad base, with elongate triangular apical part and narrow apex (Fig. 3B); aedeagus in lateral view with paramere swollen, slightly deflexed dorsad, median lobe sinuate, narrow, with fin-like apex (Fig. 3C); apex of paramere with longitudinal, median cluster of peg setae, extended basad on slight ridge (Fig. 3D). Female with tergite X narrowly elongate, with two-pronged apex, prongs separated by U-shaped emargination (Fig. 3G).

##### Distribution.

**Canada**: BC. **United States**: CA, ID, NV, OR, WA

##### Bionomics.

Smetana (1971a, b) reported this species from debris near water, especially along larger creeks and rivers. Longer series were found in river drift left behind after periods of high water levels. Several specimens have been found in beaver houses, but it is not known whether they regularly occur there.

##### Comments.

The paratype specimens mentioned by Hatch (1957) from Lenore, Idaho and Pullman, Washington were not examined but indicate this species’ distribution is rather broad across the entire Western Cordillera. The occurrence of *F.elevatus* along large river banks at a wide range of elevations suggests a single broadly distributed species. Specimens from California were paler than most of those from British Columbia and Oregon but no consistent differences were observed in the aedeagus.

## Supplementary Material

XML Treatment for Cyrtoquediini

XML Treatment for Alesiella lineipennis

XML Treatment for Sundaquedius

XML Treatment for Sundaquedius nigropolitus

XML Treatment for Sundaquedius abbreviatus

XML Treatment for Indoquediini

XML Treatment for Fluviphirus

XML Treatment for Fluviphirus elevatus
